# Aged Tendon Stem/Progenitor Cells Are Less Competent to Form 3D Tendon Organoids Due to Cell Autonomous and Matrix Production Deficits

**DOI:** 10.3389/fbioe.2020.00406

**Published:** 2020-05-05

**Authors:** Zexing Yan, Heyong Yin, Christoph Brochhausen, Christian G. Pfeifer, Volker Alt, Denitsa Docheva

**Affiliations:** ^1^Experimental Trauma Surgery, Department of Trauma Surgery, University Regensburg Medical Centre, Regensburg, Germany; ^2^Institute of Pathology, University of Regensburg, Regensburg, Germany; ^3^Department of Medical Biology, Medical University of Plovdiv, Plovdiv, Bulgaria

**Keywords:** tendon age-related degeneration, tendon stem/progenitor cells, tenogenesis, 3D cell sheet model, tendon regeneration

## Abstract

Tendons are dense connective tissues, which are critical for the integrity and function of our musculoskeletal system. During tendon aging and degeneration, tendon stem/progenitor cells (TSPCs) experience profound phenotypic changes with declined cellular functions that can be linked to the known increase in complications during tendon healing process in elderly patients. Tissue engineering is a promising approach for achieving a complete recovery of injured tendons. However, use of autologous cells from aged individuals would require restoring the cellular fitness prior to implantation. In this study, we applied an established cell sheet model for *in vitro* tenogenesis and compared the sheet formation of TSPC derived from young/healthy (Y-TSPCs) versus aged/degenerative (A-TSPCs) human Achilles tendon biopsies with the purpose to unravel differences in their potential to form self-assembled three-dimensional (3D) tendon organoids. Using our three-step protocol, 4 donors of Y-TSPCs and 9 donors of A-TSPCs were subjected to cell sheet formation and maturation in a period of 5 weeks. The sheets were then cross evaluated by weight and diameter measurements; quantification of cell density, proliferation, senescence and apoptosis; histomorphometry; gene expression of 48 target genes; and collagen type I protein production. The results revealed very obvious and significant phenotype in A-TSPC sheets characterized by being fragile and thin with poor tissue morphology, and significantly lower cell density and proliferation, but significantly higher levels of the senescence-related gene markers and apoptotic cells. Quantitative gene expression analyses at the mRNA and protein levels, also demonstrated abnormal molecular circuits in the A-TSPC sheets. Taken together, we report for the first time that A-TSPCs exhibit profound deficits in forming 3D tendon tissue organoids, thus making the cell sheet model suitable to investigate the molecular mechanisms involved in tendon aging and degeneration, as well as examining novel pharmacologic strategies for rejuvenation of aged cells.

## Introduction

Tendons are dense connective tissues, which are critical for the integrity and function of our musculoskeletal system (Schiele et al., 2013; Docheva et al., 2015; Costa-Almeida et al., 2019). Tendons are hierarchically organized and have an extracellular matrix (ECM) consisting mainly of collagen type I and a smaller portion of other collagens and proteoglycans (Yan et al., 2018). After injury, tendons need a long period for rehabilitation, especially in aged patients, accompanied with higher failure risk and unsatisfactory repair outcomes (Hirzinger et al., 2014). Tissue aging involves many intrinsic and extrinsic processes and it is frequently linked with tissue degeneration, tendon rupture incidence as well as reduced healing capacity (Gumucio et al., 2014; Schneider et al., 2018; Steinmann et al., 2020). In general, aging is reflected by a decline in organ and tissue homeostasis (de Lucas et al., 2018). Many studies on age-related diseases have revealed that a diminished stem cell pool is responsible for adult tissue degeneration (Zhang et al., 2018). However, the exact molecular mechanism behind tendon aging and degeneration remain by large unclear.

By carrying out a direct comparison between TSPCs derived from young/healthy (Y-TSPC) and aged/degenerative (A-TSPC) Achilles tendons, we have reported that A-TSPCs exhibit in two dimensional (2D) culture profound phenotypic changes with a decline in multiple cellular functions (Kohler et al., 2013). Moreover, microarray analysis showed a distinct transciptomal shift mRNA in A-TSPCs, namely, genes related to cell-cell and cell-matrix contacts, cytoskeleton and cell motility were significantly dysregulated (Kohler et al., 2013). F-actin imaging as well as quantitative kinetic analysis of cytoskeleton turnover, demonstrated that A-TSPCs have accumulation of robust actin stress fiber with slow turnover that correlated with the significantly reduced migratory and *in vitro* would healing potential of these cells (Kohler et al., 2013).

Self-assembled three-dimensional (3D) organoids, whereby cells form connections naturally between each other and to the deposited ECM, are considered as a promising culture models to investigate tissue formation *in vitro*. One widely used scaffold-free approach is the cell pellet model for *in vitro* chondrogenesis. For tenogenesis, more a tube-like cell sheet, composed of a multi-layered cellular architecture and ECM-rich patches, can be fabricated *in vitro* (Ni et al., 2013). These organoids maintain natural microenvironment and own autocrine and paracrine signaling pathways. Our recent results on 3D cell sheets formed by mesenchymal stem cells and TSPCs provided evidences for the suitability of this model to study *in vitro* tenogenic differentiation (Hsieh et al., 2018).

Thus, in this study we hypothesized that A-TSPCs will exhibit significant differences to Y-TSPCs in their potential to form 3D tendon organoids and our aims were first, to characterize the quality of the tendon sheets and second to outline dominant cellular and molecular traits underlying the expected A-TSPC phenotype.

## Materials and Methods

### Cell Culture

Primary Y-TSPCs (*n* = 4) and A-TSPCs (*n* = 9) were collected from human non-injured Achilles tendon biopsies with an average age of 28 ± 5 years and 61 ± 13 years, respectively, and extensively validated and characterized in 2D culture (Kohler et al., 2013; Popov et al., 2015) (Ethical Grant No. 166-08 of the Medical Faculty of the Ludwig-Maximilians-University, Munich). Details on donor cohort demographics, clinical indications, histological examination, inclusion and exclusion criteria are published in the Supplementary Information of Kohler et al. (2013). In short, The Y-TSPC cohort was limited to only *n* = 4 due to the rarity of such clinical samples. The donors for the A-TSPC cohort were validated for degenerative status by histological examination. For extraction and purification of the cells, the tendon tissue was minced into small pieces, digested with 0.15% collagenase II (Worthington, Lakewood, NJ, United States) enzymatically in culture medium at 37°C overnight, then filtered with sterile nylon mesh (100 μm pore size), and centrifuged at 500 *g* for 10 min. No enrichment step was implemented. Afterward, the pelleted cells were resuspended and expanded in DMEM/Ham’s F-12 medium with glutamine (365.3 mg/L), 1 × MEM amino acids, 10% FBS and 1% L-ascorbic acid-2-phosphate. Stem/progenitor character of the cells was verified in Kohler et al. (2013) by FACS and immunohistochemistry for MSC-related markers positive markers CD44, CD73, CD90, CD105, CD146 (pericyte marker), Musashi-1 and STRO-1 as well as negative markers CD19, CD34, CD45, HLA-DR) revealing a very homogeneous populations. Tendon-related genes such as the transcription factors Scleraxis, Eya1, and Six1, the tendon marker gene tenomodulin and several ECM proteins abundant in tendon (collagen types I and III, COMP, decorin, and tenascin C) were validated (Kohler et al., 2013). Self-renewal and tri-lineage differentiation assays were also carried (Kohler et al., 2013). For passaging, 60% confluent cells were detached by trypsin. Cells were used in the study at passage 2–6.

### Cell Sheet Formation

**FIGURE 1:**
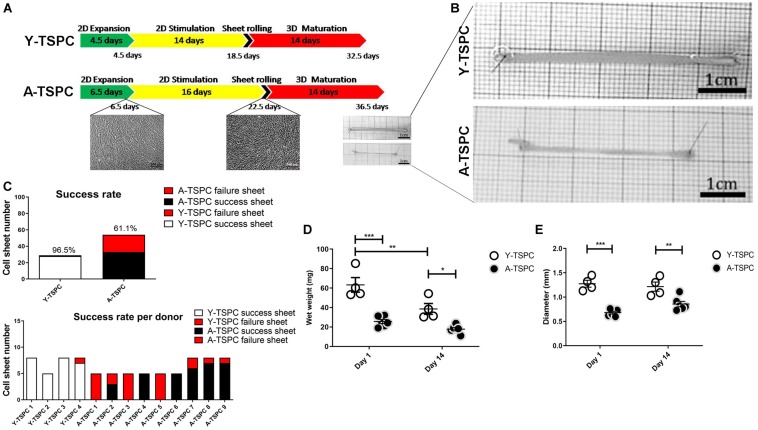
Cell sheet procedure and gross morphological data. **(A)** Cartoon depicting the 3-step protocol for cell sheet formation. **(B)** Representative macroscopy images Y-TSPC and A-TSPC sheets at day 14. **(C)** Success rate of all donors (upper panel) success rate of each individual donor (lower panel). **(D)** Comparison of TSPC sheet wet weight at day 1 and 14. **(E)** Comparison of TSPC sheet diameter at day 1 and 14. Y-TSPC *n* = 4; A-TSPC *n* = 9; *p < 0.05, **p < 0.01, and ***p < 0.001.

The cell sheet protocol, depicted in Figure 1A, comprises of a three-step procedure: expansion, stimulation and maturation (Hsieh et al., 2018). The three-step procedure is required for the self-assembly process of the cell sheet with (1) expansion – formation of confluent cell layer; (2) stimulation - for apical deposition of ECM and enrichment cell-ECM interactions (glucose for energy supply, and ascorbic acid to serve as anti-oxidant and co-factor for collagen synthesis); and (3) maturation - by tendon specific ECM production and organization in the 3D space (TGF- β3 mediated signaling is critical for tenogenesis (Havis et al., 2014, 2016). In the expansion step, both cell types (8 × 10^3^ cells/cm^2^) were cultured in 10 cm^2^ cell culture dishes (Falcon, New York, United States) until reaching full confluence in the basic medium. In the stimulation step, cells were supplemented with high glucose DMEM containing 10% FBS and 50 μg/mL ascorbic acid for 14 (Y-TSPCs) or 16.5 days (A-TSPCs). Afterwards, the continuous cell monolayers were detached manually with a cell scraper, rolled into a 3D tube-like cell sheet, stretched 10% manually and fixed with small pins in non-adhesive culture dish (Corning, New York, United States). In the last maturation step, all formed cell sheets were cultured for 14 days in maturation media containing high glucose DMEM with 10 ng/ml TGF-β3 and 50 μg/mL ascorbic acid.

### Cell Sheet Wet Weight and Diameter Analysis

Cell sheets wet weight and diameter were measured at day 1 and day 14 of the maturation step. For diameter evaluation, following algorithm was applied: (1) 5–8 sheets/donor were imaged; (2) 6 images/sheet were implemented; 5 diameters/image were measured manually and the mean was calculated; (3) the diameter of each sheet was analyzed as the mean from 6 images; (4) the diameter for each donor was expressed as mean diameter of 5–8 sheets. 4 Y-TSPC cell sheet and 9 A-TSPC cell sheet donors were applied. The quantitative data was processed with GraphPad Prism v.5 software (GraphPad software, CA, United States).

### Cell Sheet Fixation and Cryo Cutting

Y-TSPC (*n* = 4 donors) and A-TSPC (*n* = 6 donors) cell sheets (2 sheets/donor) were fixed with 4% paraformaldehyde, cryoprotected with 10, 20, 30% sucrose/phosphate-buffered saline (PBS) gradient (Sigma-Aldrich Corporation, St Louis, United States) and embedded in Tissue-Tek (Sakura Finetek, Alphen aan den Rijn, Netherlands). Cryosections (10 μm thick) were collected with cryotome (Leica, Wetzlar, Germany), and stored until use at −20°C. Prior to staining, sections were equilibrated at room temperature and rehydrated with PBS for 5 min.

### H&E (Hematoxylin and Eosin) Staining and Nuclear Angle Deviation Analysis

4 Y-TSPC (*n* = 4 donors) and 6 A-TSPC (*n* = 6 donors), the sheets (1 sheet/donor) were placed in Hematoxylin solution for 3 min, rinsed with 0.1% HCl in PBS for dedifferentiation, washed in tap water for 5 min, immersed in Eosin solution for 3 min (both from Carl Roth, Karlsruhe, Baden-Württemberg, Germany), rinsed with distilled water for 30 s and covered with Depex (Serva, Heidelberg, Germany). H&E staining images were made with Nikon digital sight DS-U camera mounted at Nikon eclipse TE2000-U microscope (Nikon, Tokyo, Japan) and used for analysis of nuclear angular deviation. Since healthy resident cells in tendon tissue are well aligned to the tensile axis (muscle to bone), this parameter is useful to assess the quality of the 3D sheet organization, and the lesser the deviation the better the alignment. The following algorithm was implemented: (1) 9 images at x20 magnification were taken randomly from each cell sheet; (2) 9 randomly chosen nuclei per image were assessed (81 nuclei/sheet); (3) the angles between the longitudinal axis of the cell sheet and the long axis of the nuclei were determined with the “angular” tool of the AxioVision software v 4.8 (Carl Zeiss, Jena, Germany); (4) 324 and 486 angles for Y- and A-TSPC cohorts were analyzed, respectively; (5) the distribution of nuclear angle deviation for each cell type was generated with GraphPad Prism.

### Nuclear Staining and Cell Density Analysis

Cryosections of Y-TSPC (*n* = 4 donors) and A-TSPC (*n* = 6 donors) sheets (1 sheet/donor) were rehydrated in water for 5 min, then stained with 4′,6-diamidino-2-phenylindole (DAPI; 1:10 × 10^3^ dilution in H2O) for 5 min at room temperature (RT) and fluoroprotected with Mowiol 4-88 (Roth, Karlsruhe, Germany). Fluorescent images were acquired at 20x magnification with an Olympus XC10 camera on an Olympus BX61 fluorescence microscope (Olympus, Japan). In brief, (1) 9 randomly taken DAPI images/sheet were analyzed with ImageJ (v1.45s software National Institutes of Health, Bethesda, MD, United States)^1^ software; (2) each image was converted into grayscale by using “threshold” adjustment tool; (3) total nuclear number and the nuclear aspect ratio (NAR) were quantified automatically with “analyze particles” tool; (4) the average cell (nuclei) density/sheet as well as per cell cohort was calculated and expressed per mm; (5) the frequency of different NAR for each cohort was generated with GraphPad Prism. Round cells are represented by NAR value close to 1, while flattened cells near to 0.

### F-Actin Staining

Cryosections of Y-TSPC (*n* = 4 donors) and A-TSPC (*n* = 6 donors) sheets (1 sheet/donor) were rehydrated in water for 5 min and permeabilizated with 0.2% Triton-X (Sigma-Aldrich) for 30 min at RT. Afterward, 1:200 phalloidin-AF488 in 1%BSA in PBS solution (Sigma-Aldrich) was applied for 60 min at RT. DAPI was used for nuclear counter-staining.

### Resazurin Assay and DNA Quantification

Resazurin assay was performed on the last day of the stimulation step according to the manufacturer’s instructions (Sigma-Aldrich). Relative immunofluorescence was measured with fluorescent ELISA reader (TECAN, Zurich, Switzerland). Y-TSPC (*n* = 3 donors) and A-TSPC (*n* = 3 donors) 2D monolayers (3 dishes/donor) were evaluated.

Cell proliferation was estimated in 3D by DNA quantification of Y-TSPC (*n* = 3 donors) and A-TSPC (*n* = 3 donors) sheets (1 sheet/donor). All samples were digested with papain for 16 h at 60°C. Next, the DNA content was quantified by using a PicoGreen DNA kit (Invitrogen, Carlsbad, CA, United States) following the manufacturer’s instructions. A standard curve based on known DNA concentration was applied to determine the total DNA content of the samples. Relative immunofluorescence was measured with TECAN.

### TUNEL (Terminal Deoxynucleotidyl Transferase dUTP Nick end Labeling) Staining and Quantification

Cell apoptosis was detected by TUNEL staining of Y-TSPC (*n* = 4 donors) and A-TSPC (*n* = 6 donors) sheets (one sheet/donor). Samples were treated with 0.2% hyaluronidase (Sigma-Aldrich) for 1 h, and then permeabilized with 0.2% Triton-X. TUNEL reaction was carried out at 37°C for 1 h. Afterward, DAPI counterstaining (1:10 × 10^3^ dilution in H_2_O) was applied and the samples were mounted for imaging. 10 μl DNase in 70 μl RNA free buffer (Qiagen, Hilden, Germany) was applied. DNA-treated samples were used as a positive control. Apoptotic cell number was measured as follow: (1) 6 images/sheet were randomly taken at x20 magnification; (2) all images were analyzed by ImageJ software; (3) apoptotic cells were counted manually and average number per donor and per cohort were expressed per mm^2^.

### RNA Isolation, cDNA Synthesis and Real Time Polymerase Chain Reaction (PCR)

Y-TSPC (*n* = 3 donors) and A-TSPC (*n* = 3 donors) sheets (1 sheet/donor) were snap frozen ad cryocrushed, then used for total RNA extraction with Qiagen RNeasy Mini kit (Qiagen) following the manufacturer’s instructions. For cDNA synthesis, 0.5 μg total RNA/sample and Transcriptor First-Strand cDNA Synthesis Kit (Roche, Mannheim, Germany) were used. Custom-designed Real-Time PCR plates with 48 different genes (Table 1) in format of 96-well/32+ by Bio-Rad (Bio-Rad Laboratories, Hercules, CA, United States) were implemented according to the manufacturer’s instructions and our previously described protocols (Yin et al., 2019). Gene expression differences were calculated with the ΔΔCT method and presented as fold change of A-TSPC group to Y-TSPC group (control). Hypoxanthine-guanine phosphoribosyltransferase 1 gene (HPRT1), low abundant but also very stable, was implemented as housekeeper in calculating ΔCT. The CT values of HPRT1 were also used as indicative of gene abundance. The average CT value of HPRT1 was 27 (*n* = 6). From the 48 target genes, 38 genes had CT values lower than HPRT1. Threshold of HPRT1 Ct (27) + 3 Ct ≥ 30 Ct was set and genes with Ct ≥ 30 were considered not detectable or very low abundant, and therefore without fold change calculation. For cell senescence analysis, Real Time PCR for p16, p21, and p53 genes (Table 2) was performed as described by Kohler et al. (2013).

**TABLE 1:** Genes included in Real Time PCR Ready Custom Designed Plates analyzed in this study.

**Target gene**	**Abbreviation**	**Category**
Early growth response 1	EGR-1	Tendon transcription factor
Early growth response 2	EGR-2	Tendon transcription factor
Eyes absent homolog 1	EYA1	Tendon transcription factor
Eyes absent homolog 2	EYA2	Tendon transcription factor
Mohawk homeobox	MKX	Tendon transcription factor
Scleraxis homolog A	SCXA	Tendon transcription factor
SIX homeobox1	SIX1	Tendon transcription factor
SIX homeobox2	SIX2	Tendon transcription factor
Collagen, type I, alpha 1	COL1A1	Collagen gene
Collagen, type III, alpha 1	COL3A1	Collagen gene
Collagen, type V, alpha 1	COL5A1	Collagen gene
Collagen, type VI, alpha 1	COL6A1	Collagen gene
Collagen, type XII, alpha 1	COL12A1	Collagen gene
Collagen, type XIV, alpha 1	COL14A1	Collagen gene
Collagen, type XV, alpha 1	COL15A1	Collagen gene
Asporin	ASPN	Collagen cross-linker gene
Lysyl oxidase	LOX	Collagen cross-linker gene
Procollagen-Lysine,2-Oxoglutarate 5-Dioxygenase 1	PLOD1, LH1	Collagen cross-linker gene
Biglycan	BGN	Tendon-related matrix gene
Decorin	DCN	Tendon-related matrix gene
Ephrin type-A receptor 4	EPHA4	Tendon-related matrix gene
Fibromodulin	FMOD	Tendon-related matrix gene
Fibronectin 1	FN1	Tendon-related matrix gene
Lumican	LUM	Tendon-related matrix gene
Proteoglycan 4	PRG4	Tendon-related matrix gene
Tenascin C	TNC	Tendon-related matrix gene
Thrombospondin 2	THBS2	Tendon-related matrix gene
Thrombospondin 4	THBS4	Tendon-related matrix gene
Tenomodulin	TNMD	Tendon-related matrix gene
Aggrecan	ACAN	Other lineage gene
Collagen, type II, alpha 1	COL2A1	Other lineage gene
Alpha-actin-2	ACTA2,**α**-SMA	Other lineage gene
Cartilage oligomeric matrix protein	COMP	Other lineage gene
Desmin	DES	Other lineage gene
Integrin-binding sialoprotein	IBSP	Other lineage gene
Fucosyltransferase 4	FUT4	Other lineage gene
Lipoprotein lipase	LPL	Other lineage gene
Myogenic differentiation 1	MYOD1	Other lineage gene
Myogenin	MYOG	Other lineage gene
Nanog homebox pseudogene 8	NANOG	Other lineage gene
Octamer-binding transcription factor 4	Oct4, Pou5f1	Other lineage gene
Peroxisome proliferator-activated receptor gamma	PPARG	Other lineage gene
Runt-related transcription factor 2	RUNX2	Other lineage gene
Transcription factor Sp7	SP7, Osterix	Other lineage gene
SRY (sex-determining region Y)-box 9	SOX9	Other lineage gene
Transcription factor AP-2 alpha	TFAP2A	Other lineage gene
Transforming growth factor beta 1	TGF-β1	Other lineage gene
Transglutaminase 2	TGM2	Other lineage gene
Beta-2-Microglobulin	B2M	Reference gene
Glyceraldehyde 3-phosphate dehydrogenase	GAPDH	Reference gene
Hypoxanthine-guanine phosphoribosyltransferase 1	HPRT1	Reference gene

**TABLE 2:** PCR for senescence-related genes p16, p21, p53.

**Target gene**	**Primers**	**Annealing temperature [°C]**	**Cycle number**	**References**
p16	F 5′-caacgcaccgaatagttacg-3′	57	35	Le Frere-Belda et al., 2004
	R 5′-agcaccaccagcgtgtc-3′			
p21	F 5′-gaacttcgactttgtcaccgag-3′	60	30	Alcantara et al., 2001
	R 5′-cgttttcgaccctgagagtctc-3′			
p53	F 5′-aaggaaatttgcgtgtggag-3′	58	35	Gan et al., 2005
	R 5′-ttctgacgcacacctattgc-3′			

### Collagen I Immunofluorescent Staining and Enzyme-Linked Immunosorbent Assay (ELISA)

Cryosections of Y-TSPC (*n* = 4 donors) and 6 A-TSPC (*n* = 6 donors) sheets (1 sheet/donor) were treated for antigen retrieval with 1% pepsin for 15 min at RT blocked by 1% bovine serum albumin (BSA) for 1 h, and incubated with primary anti-collagen type I antibody (Sigma-Aldrich, Cat. Nr. C2456, 1:200 dilution) overnight at 4°C. Next day, anti-mouse FITC secondary antibody (Sigma-Aldrich) was applied for 1h at RT, and last DAPI counterstaining was done for 5 min. Fluorescent images were acquired with an Olympus XC10 camera on an Olympus BX61 fluorescence microscope. The amount of deposited collagen I protein was detected by ELISA. 3 Y-TSPC and 5 A-TSPC donors analyzed (1 sheet/donor). First, total protein was extracted with 10 μg/ml pepsin and 1 mg/mL elastase. Next, collagen I Elisa kit (Chondrex, Redmond, WA, United States) was used according to the manufacturer’s instruction. The data was expressed as total collagen I content/DNA.

### Transmission Electron Microscopy (TEM)

Transmission electron microscopy was applied to detect the ultrastructure of Y-TSPC (*n* = 3 donors) and A-TSPC (*n* = 5 donors) cell sheets. One cell sheet per donor was prepared for TEM analyses. After rinsing with PBS, samples were fixed with Karnovsky-fixatives (0.1M cacodylate-buffer with 2.5% glutaraldehyde and 2% paraformaldehyde), then enclosed within 4% low melting agarose, post-fixed with 1% osmium tetroxide at pH 7.3, dehydrated in graded ethanol, embedded in EMbed-812 epoxy resin (Science Services, Munich, Germany) and finally polymerized for 48 h at 60°C into an EPON block. Each cell sheet was halved and embedded for longitudinal or cross-sectional cutting. Semithin sections from 0.75 μm thickness were cut and stained with toluidine blue and basic fuchsine. After selection of appropriate areas of interest, the EPON block was trimmed and ultrathin sections (80 nm thickness) were cut on Reichert Ultracut-S ultramicrotome (Leica, Bensheim, Germany). Sections were mounted on grids and stained with aqueous 2% uranyl acetate and lead citrate solution for 10 min each. Next, the sections were examined with a LEO912AB electron microscope (Zeiss, Oberkochen, Germany) operating at 100 kV. Images were taken with a side-mounted 2k x 2k-CCD-camera (TRS, Moorenweis, Germany). The number of elongated cells and apoptotic cells (images of 80 × 80 μm^2^), as well as the diameter of the collagen fibers (images of 4 × 4 μm^2^) were evaluated as follows: (1) 6 images/donor were implemented; (2) all images were analyzed by ImageJ; (3) each image was converted to grayscale by using “threshold” adjustment; (4) the number of elongated cells, apoptotic cells and collagen fibrils were counted manually and the data was expressed as the mean of counted objects per image area per donor; (5) the diameter of collagen fibrils was measured by “straight” tool manually as 20 randomly chosen fibrils per image were assessed (6 images/donor; 120 fibrils/donor; 360 fibrils for Y-TSPC group and 600 fibrils for A-TSPC group were analyzed); (6) the collagen diameter distribution per group was expressed by calculating the frequency of fibrils with different diameters (total of 360 fibrils for Y-TSPC group and total of 600 fibrils for A-TSPC group). In Figures 4, 5 representative images of Y-TSPC (*n* = 3 donors) and A-TSCP *n* = 5 (donors) are shown. GraphPad Prism v.5 software was used for quantitative data analyses and graphical data expression.

### Statistics

GraphPad Prism v.5 software was used for expressing quantitative data and estimation of statistical significance. Unless distribution and frequency data, all data shows individual donors (dot plot) and mean values and standard deviations for each cohort. Statistical testing was performed with unpaired *t*-test. Difference were considered statistically significant when ^∗^*p* < 0.05, ^∗∗^*p* < 0.01, and ^∗∗∗^*p* < 0.001.

## Results

### A-TSPC Showed Inferior Sheets Formation With Higher Failure Rate

In the first expansion step, A-TSPCs needed 2 more days to reach full cell confluence than Y-TSPCs. In the stimulation step, Y-TSPCs took 14 days to form a cell sheet, whilst A-TSPCs required 16.5 days (Figure 1A). The gross appearances of sheets from both cell types were evaluated and compared (Figure 1B), showing that A-TSPC sheets were much thinner and smaller than Y-TSPC sheets. Then, as mentioned in the Materials and Methods, 4 Y-TSPC donors and 9 A-TSPC donors were used in the study, and the total success rate for Y-TSPC sheet formation was 96.5% with only 1 failed sheet from Y3-TSPC donor (ruptured) because of the over stretching when handled. In contrast, for the A-TSPC group the overall success rate of sheet formation was only 61.1% and certain donors namely A1-, A3-, and A5-TSPC being unable to form any sheet (Figure 1C). Next, cell sheet diameter and wet weight at day 1 and 14 were measured confirming that A-TSPC sheets were significantly smaller and lighter than Y-TSPC sheets at both the time points (Figures 1D,E).

### A-TSPC Sheets Had Lower Cell Density, Disorganized Matrix and Poor Cell Alignment

**FIGURE 2:**
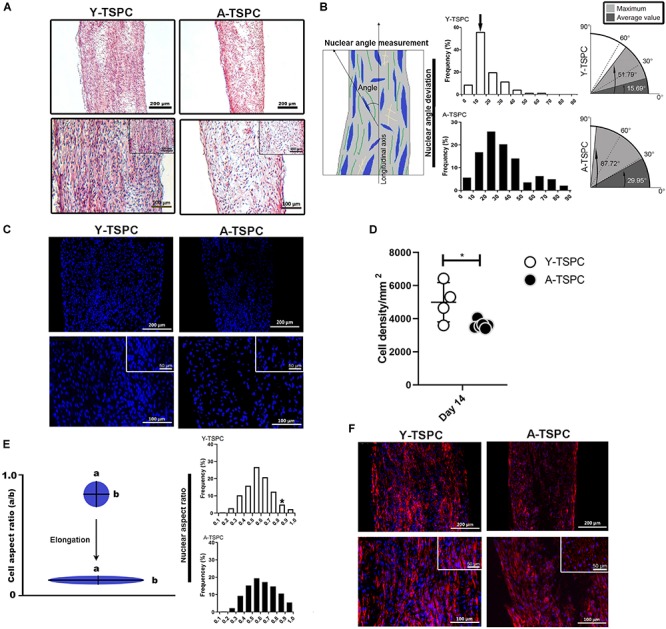
Y-TSPC and A-TSPC histomorphometrical analyses. **(A)** Representative H&E images of Y-TSPC and A-TSPC sheets at day 14. **(B)** Frequency distribution of nuclear angle deviation (left panel) as well as maximum and average values of angular deviation (right panel, virtual angle between cell sheet longitudinal axis and nuclear axis). The arrow indicates the higher frequency of nuclear deviation of 10°–20° deviation in the Y-TSPC sheets. **(C)** Representative images of nuclear staining via DAPI (blue color) of Y-TPSC and A-TPSC sheets. **(D)** Average cell density of both groups. **(E)** Frequency distribution of NAR (nuclear aspect ratio, nucleus width versus length). The arrow indicates the higher tendency of NAR 0.8-1 (round cells) in A-TSPC group. (F) Representative F-actin images of Y-TSPC and A-TSPC sheets at day 14. Y-TSPC *n* = 4; A-TSPC *n* = 6; *p < 0.05*.

The general tissue morphology of the cell sheets was revealed by H&E staining (Figure 2A) and analysis of the angle of nuclear deviation (Figure 2B). A-TSPC cells formed an inferior cell sheet with less matrix deposition and frequent gaps. Y-TSPC formed bigger cell sheet containing a large amount of aligned fibrous matrix with higher number of spindle-like shaped cells. In addition, the cells in the A-TSPC sheets were sparser and rounded compared to the Y-TSPC group. Cell orientation was assessed by estimating the angular deviation of the cell nuclei to the axial axis of the cell sheet. The results showed that in the Y-TSPC group, over 60% of the nuclei deviated 0° to 20° from the axial axis, and the average nuclear angle was 15.69°. In contrast, in the A-TSPC group, less than 30% of the nuclei deviated from 0° to 20°, and the average nuclear deviation was nearly twofold increased to 29.95°. Next, the cell density and frequency distribution of the nuclei aspect ratio, which represents the nuclei flattening, were quantified (Figures 2C–E) and revealed a significantly lower cell density in the aged group. Moreover, A-TSPC sheets contained significantly higher frequency of cells with NAR 0.8-0.9 corresponding to roundish cells. Analyses of the cell cytoskeleton via phalloidin fluorescent staining confirmed worse cell elongation and cytoskeletal organization in the A-TSPC group than the Y-TSPC group (Figure 2F).

### A-TSPC Sheets Showed Reduced Metabolic and Proliferative Activities, Whilst Augmented Apoptosis, Senescence

**FIGURE 3:**
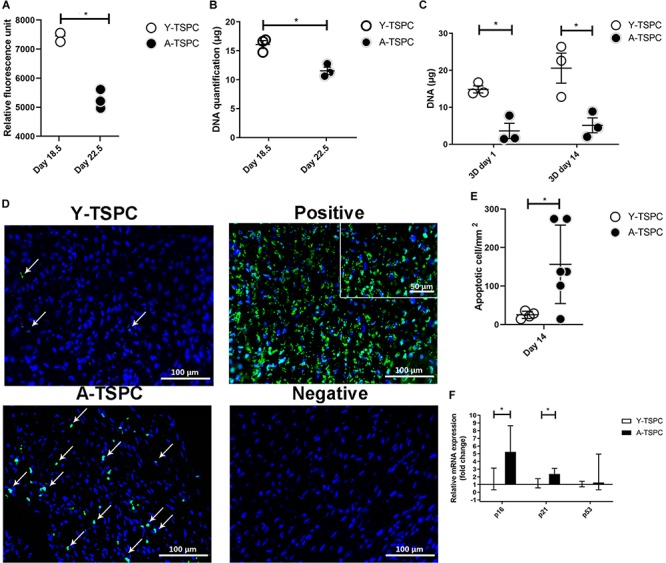
Quantitative analyses of cell proliferation, apoptosis and senescence. **(A,B)** Resazurin assay and DNA quantification at 2D prior sheet collection. **(C)** DNA quantification at 3D day 1 and day 14. **(D)** Representative TUNEL/DAPI images at 3D day 14. Apoptotic cells appear in green. Positive control with DNase treated. Negative control with label solution only (without terminal transferase). **(E)** Quantification of average apoptotic cell number in the sheets at day 14. **(F)** Quantitative PCR analysis of p16, p21, and p53. Y-TSPC *n* = 4; A-TSPC *n* = 6; *p < 0.05 and ***p < 0.001. For PCR, Y-TSPC *n* = 3; A-TSPC *n* = 3.

For cell proliferation, Resazurin assay was carried out at the 2D cell monolayer level prior to sheet formation (day 18.5 for Y-TSPCs and day 22.5 for A-TSPC), followed by DNA content quantification. DNA was also quantified at the 3D cell sheet level at day 1 and day 14 (Figures 3A–C). The results showed that A-TSPC group contained significantly lower cell amount than Y-TSPC group, paralleled by significantly higher metabolic cell activity, in the 2D step. At the 3D level, the data suggested that after the collection of the cell sheets in the A-TSPC group there is further cell loss. In contrast, in the period day 1 to day 14 at the 3D level cell proliferation occurred in the Y-TSPC sheets as indicated by an increase in DNA content. Furthermore, TUNEL staining of Y-TSPC and A-TSPC cell sheets revealed that A-TSPC sheets have significantly more apoptotic cells than Y-TSPC sheets (Figures 3D,E). Cell senescence analysis was performed by quantitative PCR for the expression of cell cycle regulator genes p16, p21, and p53 and it demonstrated a significant upregulation of p16 and p21 in the A-TSPC sheet group (Figure 3F).

### A-TSPC Sheets Exhibited a Profound Phenotype at the Ultrastructural Level

**FIGURE 4:**
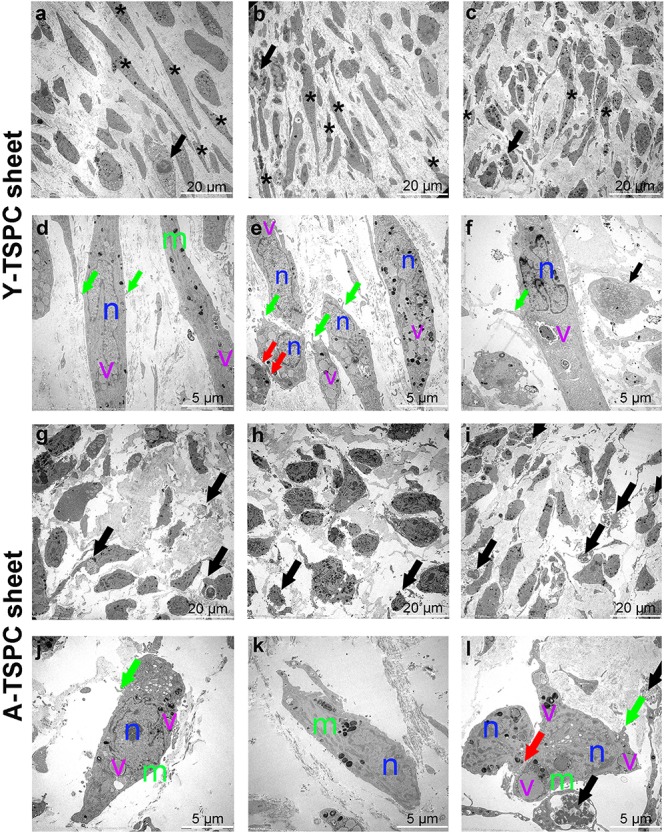
Representative transmission electron microscopy (TEM) images of Y-TSPC and A-TSPC sheets. **(a–c)** Y-TSPC sheet cell arrangement, cell density and morphology. **(d–f)** Close view of Y-TSPC morphology and cell protrusions. **(g–i)** A-TSPC sheet cell arrangement, cell density and morphology. **(j–l)** Close view of A-TSPC morphology and cell protrusions. Black arrows, apoptotic cells; green arrows, cell protrusions; red arrows, cell–cell contact; m, mitochondria; n, nucleus; v, vacuoles; *, longitudinal cell; Y-TSPC *n* = 3, A-TSPC *n* = 5 were investigated.

**FIGURE 5:**
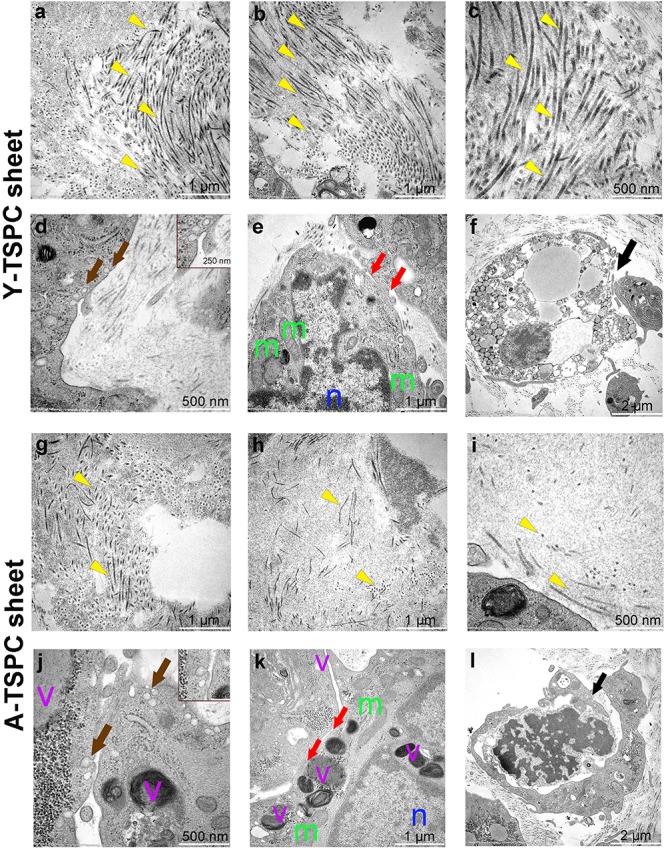
Representative transmission electron microscopy (TEM) images of Y-TSPC and A-TSPC sheets. **(a–c)** Y-TSPC sheet collagen fibrils. **(d)** Y-TSPC sheet plasmalemma vesicles at the cell membrane. **(e)** Y-TSPC sheet cell-to-cell contacts between cells. **(f)** Y-TSPC sheet close view of apoptotic bodies. **(g–i)** A-TSPC sheet collagen fibrils. **(j)** A-TSPC sheet plasmalemma vesicles at the cell membrane. **(k)** A-TSPC sheet cell-to-cell contacts between cells. **(l)** A-TSPC sheet close view of apoptotic bodies. Arrow head, collagen fibrils; black arrows, apoptotic cells, brown arrows, plasmalemma vesicles; red arrows, cell–cell contact; m, mitochondria; n, nucleus; v, vacuoles; Y-TSPC *n* = 3, A-TSPC *n* = 5 were investigated.

**FIGURE 6:**
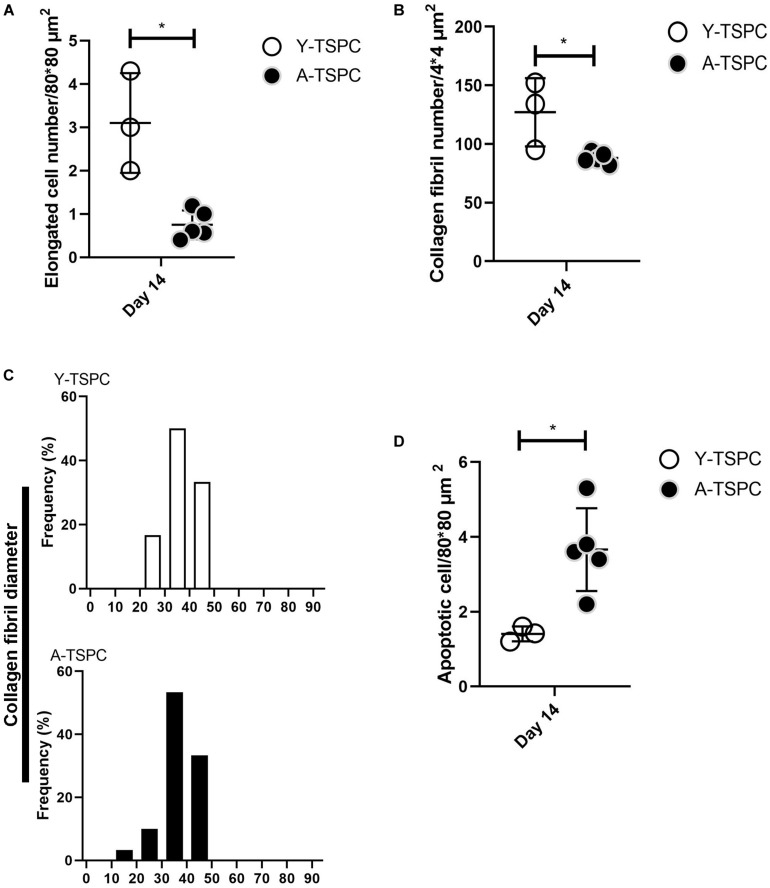
Quantification of elongated cells **(A)**, collagen fibrils and diameter distribution **(B,C)** and apoptotic cell numbers **(D)** from TEM images. Y-TSPC *n* = 3; A-TSPC *n* = 5; for **(A,B,D)**, 6 images/donor were analyzed; for **(C)** 20 randomly chosen fibrils per image were assessed; *p < 0.05.

The ultrastructure of Y-TSPC and A-TSPC sheets was revealed by TEM. In Y-TSPC sheets, cells appeared elongated and in many locations organized in parallel rows compared to A-TSPC sheets (Figures 4a–l). In addition, Y-TSPC sheets had higher cellular density (Figures 4a–c); the ECM was rich in collagen type I fibrils that were densely packed (Figures 5a–c). In contrast, A-TSPCs exhibited a rounded morphology within the sheets as well as their ECM was less dense and contained few sparsely distributed collagen fibrils (Figures 4g–l, 5g–i). In the Y-TSPC sheets, plasmalemma vesicles at the cell membrane (Figure 5d) as well as cell protrusions (Figures 4d–f) and cell-cell contacts (Figure 5e) were frequently detected. They were also visible in A-TSPC sheets but very rarely (Figures 4j–l, 5j,k). Interestingly, A-TSPC sheets contained multiple apoptotic bodies indicative of cells that undergo programmed cell death (Figures 4g–i, 5l). Apoptotic cells were rare in sheets formed by Y-TSPCs (Figures 4a–c, 5f). However, both types of cells however were comparable regarding vacuoles and mitochondria (Figures 4d–f,j–l, 5d,e,j,k). Next, quantification analysis of TEM image data confirmed the above observation and clearly showed that A-TSPC sheets had a significantly reduced number of elongated cells and collagen fibrils, but significantly increased number of apoptotic cells (Figures 6A,B,D). Regarding collagen fibril diameter distribution, both types of cell sheet were comparable with fibril diameter spanning 10–50 nm (Figure 6C). In all, the TEM analyses strongly confirmed the profound phenotype of the A-TSPC sheet at the ultrastructural level.

### A-TSPC Sheets Contained Lower Collagen I Protein

**FIGURE 7:**
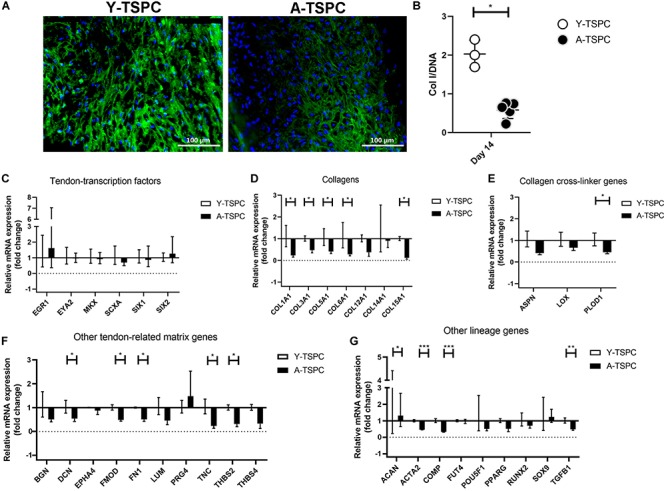
Collagen I protein production and gene expression profiling by Real Time PCR. **(A)** Representative collagen I immunofluorescent images of Y-TSPC and A-TSPC sheets at day 14. **(B)** Quantitative analysis of collagen I by ELISA. For **(A,B)** Y-TSPC *n* = 3; A-TSPC *n* = 6. Normalization to DNA. Quantitative PCR analysis for tendon-transcription factors **(C)**, collagen genes **(D)**, cross-linker genes **(E)**, tendon-related ECM genes **(F)** other lineage genes **(G)**. Gene expression was calculated as fold change to Y-TSPC group. GAPDH was used as reference gene in each group. Non-detectable (Ct over 38 genes were not included in the graphs. For **(C–G)** Y-TSPC *n* = 3; A-TSPC *n* = 3; ^∗^*p* < 0.05, ^∗∗^*p* < 0.01, and ^∗∗∗^*p* < 0.001.

In addition, to the TEM analysis, collagen type one deposition was further validated by conducting collagen I immunofluorescence staining and ELISA (Figures 7A,B). Our results clearly demonstrated that A-TSPC sheets had significantly lower collagen I protein content than Y-TSPC sheets.

### A-TSPC Sheets Displayed Abnormal Gene Expression Profile

Real-time PCR for 48 different genes (Table 1) was carried out with mRNA/cDNA collected from Y-TSPC and A-TSPC sheets at day 14. Gene markers for other lineages, namely for cartilage - collagen 2a1 (COL2A1) (cartilage); for muscle – desmin (DES), myogenic differentiation 1 (MYOD1) and myogenin (MYOG); for bone – Sp7 (SP7, Osterix) and integrin-binding sialoprotein (IBSP); for fat – AP-2 alpha (TFAP2A) and lipoprotein lipase (LPL); and for embryonic lineage – Nanog homebox pseudogene 8 (NANOG) were not detectable. Several genes that are tendon-related – early growth response protein 2 (EGR2), eyes absent homolog 1 (EYA1), transglutaminase 2 (TGM2) and tenomodulin (TNMD) were also not detectable or very low abundant. The mRNA levels of tendon transcription factors (Figure 7C) were comparable between the two groups. Several collagen genes namely collagen 1a1 (COL1A1), collagen 3a1 (COL3A1), collagen 5a1 (COL5A1), collagen 6a1 (COL6A1), collagen 15a1 (COL15A1), had significantly lower expression in the A-TSPC sheets (Figure 7D). In addition, a gene related to collagen cross-linking such as procollagen-lysine, 2-oxoglutarate 5-dioxygenase 1 (PLOD1) and the matrix proteoglycans decorin (DCN), fibromodulin (FMOD), fibronection (FN), tenascin C (TNC) and thrombospondin 2 were also significantly downregulated in the A-TSPC sheets (Figures 7E,F). Regarding other lineage related genes, three genes aggrecan (ACAN), alpha-actin 2 (ACTA2), cartilage oligomeric matrix protein (COMP) were also found to be expressed to a lower extent in the A-TSPC group (Figure 7G). Altogether, this data demonstrated a fundamental matrix synthesis problem in the A-TSPC sheets.

## Discussion

Tendon injuries, which associate closely with aged-related tissue degeneration present great challenges to orthopedic and trauma surgery departments. Aged-related tissue degeneration has been previously linked to impaired functions of tissue-resident stem/progenitor cells (Smith et al., 2002; Rees et al., 2006; Asai et al., 2014). Han et al. suggested that with donor aging smooth muscle-derived MSCs have greatly diminished self-renewal and proliferation abilities but augmented senescence (Han et al., 2010). In order to investigate how aging affects TSPCs, we compared the phenotypic difference between Achilles tendon-derived Y-TSPC from young/healthy and A-TSPC from aged/degenerative human donors. Our previous data in 2D culture, revealed that A-TSPC had a significantly reduced cell adhesion, migration and self-renewal abilities with distinct clonogenic deficits and premature entry into senescence (Kohler et al., 2013), suggesting that the TSPC pool is becoming exhausted in terms of size and functional fitness during tendon age-related degeneration.

Here, we applied a previously established cell sheet model (Hsieh et al., 2018) for *in vitro* tenogenesis and compared the sheet formation of Y-TSPCs and A-TSPCs with the purpose of unraveling differences in their potential to form 3D tendon organoids. Formation of cell sheets is an alternative scaffold-free approach to build an abundant ECM niche without elimination of the native connections between cells to cells and cells to ECM. Furthermore, the cell multilayer contains a natural microenvironment and maintains its own autocrine and paracrine signaling (Hsieh et al., 2018). Several growth factors such as members of the Transforming Growth Factor beta family, TGF-β1, β2, and β3, have been reported to be critical for tendon tissue formation, function and healing (Pryce et al., 2009; Maeda et al., 2011; Havis et al., 2014; Tan et al., 2020). Connective Tissue Growth Factor (CTGF) has also been linked to tenogenesis; for example, it is pro-tenogenic factor of adipose-derived stem cells (Shen et al., 2018; Li et al., 2019). Interestingly, Yin et al. (2016) have combined CTGF and TGF-β1 in a step-wise manner, which led to augmented tenogenic differentiation of MSCs, as well as to tendon-like tissue formation in the repair process of rat patella tendon defect. CTGF alone or in combination with TGF-β has also been associated with fibrocartilage formation in meniscal injury models (Lee et al., 2014; Tarafder et al., 2019). Therefore, as well as to minimize costs, we designed our study with administration of only one growth factor from the TGFβ family. However, it will be of great importance in follow up research to investigate the synergistic action of CTGF and TGFβ in the 3D tendon cell sheet model, which may result in quicker maturation of the cell sheets.

In the present study, our results clearly showed that A-TSPCs exhibit profound deficits in forming 3D tendon tissue organoids. There are several possible explanations: A-TSPC sheets have (1) reduced cell proliferation; (2) augmented cell apoptosis and senescence; and (3) less ECM production.

Tissue and organ aging has a strong relationship with stem cell proliferation ability, apoptosis and senescence. Kasper et al. (2009) reported that the size of MSCs pool reduces in aged animals, compared to young animals. Yu et al. (2011) demonstrated that aged rhesus macaque BMSCs require longer population doubling time due to decreased proliferation capacity. In our study, cell proliferation was tested prior to sheet collection and at day 1 and 14, revealing that A-TSPC sheets have significantly lower cell density and proliferation rate than Y-TSPC sheets. Kawakami et al. (2019) communicated that progeroid mice contained higher numbers of apoptotic muscle stem/progenitor cells. Taylor et al. (2003) showed that in human colonic crypts, age-related DNA mutations are associated with stem cell apoptosis. By performing TUNEL assays for DNA damage, we observed that the A-TSPC sheets also contain more apoptotic cells than the Y-TSPC group. Furthermore, we found that senescence-related gene markers namely p16 and p21 (Campisi, 2005) were significantly upregulated in the A-TSPC group, This finding is in line with skeletal muscle aging, where local stem cells undergo a dysregulation in p16 signaling that is directly related to an augmentation in muscle tissue senescence. Interestingly, reducing p16 expression might be a way to postpone tissue and organ aging (Baker et al., 2011). Silencing of p16 expression can reduce the number of senescent cells thus restoring the regenerative potential of muscle tissue, which was shown by Sousa-Victor et al. (2014). In all, the above results indicated that A-TSPC 3D sheets, characterized with a lower cell density and proliferation potential, whilst having higher cell apoptosis and senescence, are significantly inferior then Y-TSPC sheets.

Tendon ECM is very fibrous and organized in parallel collagen fibers. Roughly 95% of the collagen is type I, with small levels of collagen types III, V, VI, XII, XIV, and XV. The non-collageneous proteins in the tendon ECM belong to proteoglycan and glycoprotein family and smaller amount of proteoglycans (e.g., decorin, biglycan, fibromodulin, lumican) such glycoproteins (e.g., COMP, TNC, PRG4. TNMD) (Docheva et al., 2015; Screen et al., 2015). In the current study, we could not detect TNMD gene expression in neither of the TSPC groups, most likely due to transcript loss in the cell expansion step. Rapid downregulation of TNMD mRNA in 2D and 3D cultured human TSPCs was previously described (Dex et al., 2017). The ECM provides a natural physical microenvironment for cellular connections and also regulates cellular processes such as proliferation and differentiation (Theocharis et al., 2016). Aged stem cells still possess multipotency and are capable of ECM deposition (Stolzing and Scutt, 2006; Yu et al., 2011). However, it has been reported that cell aging can lead to a reduction in COL content (Haut et al., 1992; Couppe et al., 2009). In our study, we found that A-TSPC sheets contained significantly less ECM with poor alignment when compared to the Y-TSPC sheets. Connor et al. (2019) showed that senescent fibroblast has downregulated COL1A1 and elastin gene expression, compared to young fibroblast. This is comparable to our study revealing in A-TSPC sheets had a significant lower COL1 expression at mRNA and protein levels. COL 3 and COL 5, fibrillar collagens expressed in the human tendon tissue, that are important for the optimal fibrillary formation and tissue quality (Kannus, 2000) were both also significantly downregulated in the A-TSPC group. Our results are in line with two omics studies. Proteomic analysis of engineered tendon constructs by young or aged equine tenocytes revealed that tendon aging was associated with altered ECM protein, and especially COL type I, III and IV as well as proteins involved in cell-matrix interaction and mechanotransduction such as calponin 1, paladin, caldesmon 1 and contracting (Turlo et al., 2018). Moreover, the data suggested a lower cytoskeleton turnover in the aged group (Turlo et al., 2018), confirming independently the findings in Kohler et al. (2013). Transcriptome comparison of uninjured human Achilles tendon tissue biopsies from young and old patients demonstrated with tendon age a differential expression of genes related to cellular function, growth and the cell cycle (Peffers et al., 2015). Interestingly, downregulation of COL transcripts and genes involved in the post translational modification of COL were also affected (Peffers et al., 2015). A gene called SPARC (secreted protein acidic and rich in cysteine) has been shown to play an important role in ECM assembly and particularly in dermal fibroblast to regulate processing of procollagen I and COL fibrilogenesis (Rentz et al., 2007; Bradshaw, 2009). Interestingly, tendon tissues from Sparc-deficient mice, also exhibited altered actin cytoskeleton as well as collagen fibrilogenesis defects (Gehwolf et al., 2016) and hence, will be relevant to assess in the future, SPARC expression levels in the A-TSPC sheets. On the contrary, the study by Couppe et al. (2014) suggested that the COL fibril numbers remain unchanged during aging in both animal and human tendon tissue, but the enzymatic or non-enzymatic cross-linkers of the fibrils, thereby their size, can be affected during aging (Svensson et al., 2016). We analyzed other genes related to COL fibril regulation such as proteoglycans (DCN, FMOD, FN1, TNC, THBS2, COMP) and PLOD1, and found several being significantly downregulated in the A-TSPC group. These novel molecular candidates can be the foundation in follow up studies to investigate molecular cascades in both directions – up and downstream in order to elucidate the molecular mechanisms leading to the deficiency in matrix synthesis by A-TSPCs. Altogether, this data indicates the A-TSPC group harbors significant ECM production deficit.

With aging some structural changes of the COL fibrils occur; as mentioned above, changes in their size, but also shape and even biomechanical properties (Rahmati et al., 2017). We carried out TEM and investigated COL fibril diameter size but found no significant differences between the groups. One possibility is that during *in vitro* culture without dynamic mechanical loading the lateral growth of the COL fibril size is limited. In future, it will be of interest to subject both type of sheets to periodic axial stretching, as well as to examine their biomechanical properties, for example by the means of Force Indentation Atomic Force Microscopy. Nevertheless, the TEM analyses independently validated that A-TSPC contained less and disorganized cells exhibiting round and apoptotic morphologies embedded in a very poor COL matrix. The structural and biomechanical properties of the ECM can profoundly affect cell behavior. Interestingly, providing A-TSPCs with hydrogel environment consisting of aligned nanonfibers alleviated their aged phenotype (Yin et al., 2020). Hence, “engineering a healthy” ECM niche might be an attractive way to rejuvenate aged cells, support their growth and direct their fate decision and differentiation.

Our study has several shortcomings that need to be addressed in follow up research: (1) Due to the manual rolling of the cell sheets, some ECM and cells are lost, therefore standardizing this process will be of importance; (2) A time point longer that 14 days can be evaluated to investigate if the A-TSPC sheets further deteriorate; (3) As mentioned above, subjecting the 3D tendon organoids to periodic axial stretching will be relevant to examine if this can lead to sheet diameter growth. In addition, efforts to speed up the sheet formation process and to become more economic, regarding required cell numbers will be very important.

Taken together, our study reports as in 2D culture, so in 3D culture A-TSPCs show cell autonomous deficits namely, significantly reduced renewability but significantly increased senescence and apoptosis. Furthermore, we report the following novel findings: (1) A-TSPCs have a severe issue with ECM production, which was validated at the mRNA and protein levels; (2) they are less competent to organize the ECM and to properly align in the third dimension; (3) Q-PCR profiling identified novel molecular candidates such as collagen genes (COL I, III, V, VI, and XV) and proteoglycans (DCN, FMOD, FN1, TNC, THBS2, COMP, PLOD1) which can help to elucidate the exact molecular circuits behind the deficiency in ECM synthesis. Further validation at the protein level as well as “gain” and “loss” of function experiments will be of great interest to follow. Last but not least, the 3D sheet model can become very attractive for (1) developing an effective 3D tenogenic differentiation protocol for wide use similarly to the well-known and accepted chondorgenic in pellet cultures and (2) for screening of small pharmacological cues for rejuvenation of the A-TSPC sheets and to evaluate side effects of available or in development medications on tendon tissues.

## Data Availability Statement

The datasets generated for this study are available on request to the corresponding author.

## Ethics Statement

The studies involving human participants were reviewed and approved by Ethics Committee of the Medical Faculty of the Ludwig-Maximilians-University, Munich (Ethical Grant No. 166-08). The patients/participants provided their written informed consent to participate in this study.

## Author Contributions

DD conceived and designed the study, analyzed the data, and wrote the manuscript. ZY designed and performed the experiments, analyzed the data, and wrote the manuscript. HY analyzed the data and edited the manuscript. CB conducted TEM experiments. CP and VA provided the clinical expertise. All co-authors have read and approved the manuscript.

## Conflict of Interest

The authors declare that the research was conducted in the absence of any commercial or financial relationships that could be construed as a potential conflict of interest.

## Footnotes

1 https://imagej.nih.gov/ij/

## Abbreviations

ACAN: aggrecan
ACTA2: alpha-actin 2
ADSC: adipose-derived stem cell
AR: aspect ratio
A-TSPC: aged tendon stem/progenitor cell
BMSC: bone mesenchymal stem cell
BSA: bovine serum albumin
CDKN2a: cyclin-dependent kinase Inhibitor 2a
COL: collagen
COMP: cartilage oligomeric matrix protein
DCN: decorin
DES: desmin
ELISA: enzyme-linked immunosorbent assay
ECM: extracellular matrix
FMOD: fibromodulin
FN: fibronectin
LPL: lipopretionlipase
NAR: nuclear aspect ratio
OSX: osterix
PBS: phosphate buffered saline
PLOD: procollagen-Lysine,2-Oxoglutarate 5-Dioxygenase 1
RT: room temperature
TEM: transmission electron microscopy
TGFB1: transforming growth factor beta 1
THBS: thrombospondin
TNC: tenascin C
TUNEL: terminal deoxynucleotidyl transferase dUTP nick end labeling
Y-TSPC: young tendon stem/progenitor cell.

## References

[B1] Alcantara O. Kalidas M. Baltathakis I. Boldt D. H. (2001). Expression of multiple genes regulating cell cycle and apoptosis in differentiating hematopoietic cells is dependent on iron. *Exp. Hematol.* 29 1060–1069. 10.1016/s0301-472x(01)00683-x 11532346

[B2] Asai S. Otsuru S. Candela M. E. Cantley L. Uchibe K. Hofmann T. J. (2014). Tendon progenitor cells in injured tendons have strong chondrogenic potential: the CD105-negative subpopulation induces chondrogenic degeneration. *Stem Cells* 32 3266–3277. 10.1002/stem.1847 25220576 PMC4245375

[B3] Baker D. J. Wijshake T. Tchkonia T. LeBrasseur N. K. Childs B. G. van de Sluis B. (2011). Clearance of p16Ink4a-positive senescent cells delays ageing-associated disorders. *Nature* 479 232–236. 10.1038/nature10600 22048312 PMC3468323

[B4] Bradshaw A. D. (2009). The role of SPARC in extracellular matrix assembly. *J. Cell Commun. Signal.* 3 239–246. 10.1007/s12079-009-0062-6 19798598 PMC2778582

[B5] Campisi J. (2005). Senescent cells, tumor suppression, and organismal aging: good citizens, bad neighbors. *Cell* 120 513–522. 10.1016/j.cell.2005.02.003 15734683

[B6] Costa-Almeida R. Calejo I. Gomes M. E. (2019). Mesenchymal stem cells empowering tendon regenerative therapies. *Int. J. Mol. Sci.* 20:3002. 10.3390/ijms20123002 31248196 PMC6627139

[B7] Couppe C. Hansen P. Kongsgaard M. Kovanen V. Suetta C. Aagaard P. (2009). Mechanical properties and collagen cross-linking of the patellar tendon in old and young men. *J. Appl. Physiol.* 107 880–886. 10.1152/japplphysiol.00291.2009 19556458

[B8] Couppe C. Svensson R. B. Grosset J. F. Kovanen V. Nielsen R. H. Olsen M. R. (2014). Life-long endurance running is associated with reduced glycation and mechanical stress in connective tissue. *Age* 36:9665. 10.1007/s11357-014-9665-9 24997017 PMC4150896

[B9] de Lucas B. Perez L. M. Galvez B. G. (2018). Importance and regulation of adult stem cell migration. *J. Cell Mol. Med.* 22 746–754. 10.1111/jcmm.13422 29214727 PMC5783855

[B10] Dex S. Alberton P. Willkomm L. Sollradl T. Bago S. Milz S. (2017). Tenomodulin is required for tendon endurance running and collagen I fibril adaptation to mechanical load. *EBioMedicine* 20 240–254. 10.1016/j.ebiom.2017.05.003 28566251 PMC5478207

[B11] Docheva D. Muller S. A. Majewski M. Evans C. H. (2015). Biologics for tendon repair. *Adv. Drug Deliv. Rev.* 84 222–239. 10.1016/j.addr.2014.11.015 25446135 PMC4519231

[B12] Gan L. Yang X. L. Liu Q. Xu H. B. (2005). Inhibitory effects of thioredoxin reductase antisense RNA on the growth of human hepatocellular carcinoma cells. *J. Cell. Biochem.* 96 653–664. 10.1002/jcb.20585 16088946

[B13] Gehwolf R. Wagner A. Lehner C. Bradshaw A. D. Scharler C. Niestrawska J. A. (2016). Pleiotropic roles of the matricellular protein Sparc in tendon maturation and ageing. *Sci. Rep.* 6:32635. 10.1038/srep32635 27586416 PMC5009305

[B14] Gumucio J. P. Korn M. A. Saripalli A. L. Flood M. D. Phan A. C. Roche S. M. (2014). Aging-associated exacerbation in fatty degeneration and infiltration after rotator cuff tear. *J. Shoulder Elbow. Surg.* 23 99–108. 10.1016/j.jse.2013.04.011 23790676 PMC3785561

[B15] Han J. Liu J. Y. Swartz D. D. Andreadis S. T. (2010). Molecular and functional effects of organismal ageing on smooth muscle cells derived from bone marrow mesenchymal stem cells. *Cardiovasc. Res.* 87 147–155. 10.1093/cvr/cvq024 20097675 PMC2883893

[B16] Haut R. C. Lancaster R. L. DeCamp C. E. (1992). Mechanical properties of the canine patellar tendon: some correlations with age and the content of collagen. *J. Biomech.* 25 163–173. 10.1016/0021-9290(92)90273-4 1733992

[B17] Havis E. Bonnin M. A. Esteves de Lima J. Charvet B. Milet C. Duprez D. (2016). TGFbeta and FGF promote tendon progenitor fate and act downstream of muscle contraction to regulate tendon differentiation during chick limb development. *Development* 143 3839–3851. 10.1242/dev.136242 27624906

[B18] Havis E. Bonnin M. A. Olivera-Martinez I. Nazaret N. Ruggiu M. Weibel J. (2014). Transcriptomic analysis of mouse limb tendon cells during development. *Development* 141 3683–3696. 10.1242/dev.108654 25249460

[B19] Hirzinger C. Tauber M. Korntner S. Quirchmayr M. Bauer H. C. Traweger A. (2014). ACL injuries and stem cell therapy. *Arch. Orthop. Trauma Surg.* 134 1573–1578. 10.1007/s00402-014-2060-2 25073617

[B20] Hsieh C. F. Yan Z. Schumann R. G. Milz S. Pfeifer C. G. Schieker M. (2018). In vitro comparison of 2D-Cell culture and 3d-cell sheets of scleraxis-programmed bone marrow derived mesenchymal stem cells to primary tendon stem/progenitor cells for tendon repair. *Int. J. Mol. Sci.* 19:2272. 10.3390/ijms19082272 30072668 PMC6121892

[B21] Kasper G. Mao L. Geissler S. Draycheva A. Trippens J. Kuhnisch J. (2009). Insights into mesenchymal stem cell aging: involvement of antioxidant defense and actin cytoskeleton. *Stem Cells* 27 1288–1297. 10.1002/stem.49 19492299

[B22] Kawakami Y. Hambright W. S. Takayama K. Mu X. Lu A. Cummins J. H. (2019). Rapamycin rescues age-related changes in muscle-derived stem/progenitor cells from progeroid mice. *Mol. Ther. Methods Clin. Dev.* 14 64–76. 10.1016/j.omtm.2019.05.011 31312666 PMC6610712

[B23] Kohler J. Popov C. Klotz B. Alberton P. Prall W. C. Haasters F. (2013). Uncovering the cellular and molecular changes in tendon stem/progenitor cells attributed to tendon aging and degeneration. *Aging Cell* 12 988–999. 10.1111/acel.12124 23826660 PMC4225469

[B24] Lee C. H. Rodeo S. A. Fortier L. A. Lu C. Erisken C. Mao J. J. (2014). Protein-releasing polymeric scaffolds induce fibrochondrocytic differentiation of endogenous cells for knee meniscus regeneration in sheep. *Sci. Transl. Med.* 6:266ra171. 10.1126/scitranslmed.3009696 25504882 PMC4546837

[B25] Le Frere-Belda M. A. Gil Diez de Medina S. Daher A. Martin N. Albaud B. Heudes D. (2004). Profiles of the 2 INK4a gene products, p16 and p14ARF, in human reference urothelium and bladder carcinomas, according to pRb and p53 protein status. *Hum. Pathol*. 35, 817–824. 10.1016/j.humpath.2004.01.019 15257544

[B26] Li X. Pongkitwitoon S. Lu H. Lee C. Gelberman R. Thomopoulos S. (2019). CTGF induces tenogenic differentiation and proliferation of adipose-derived stromal cells. *J. Orthop. Res.* 37 574–582. 10.1002/jor.24248 30756417 PMC6467286

[B27] Maeda T. Sakabe T. Sunaga A. Sakai K. Rivera A. L. Keene D. R. (2011). Conversion of mechanical force into TGF-beta-mediated biochemical signals. *Curr. Biol.* 21 933–941. 10.1016/j.cub.2011.04.007 21600772 PMC3118584

[B28] Ni M. Rui Y. F. Tan Q. Liu Y. Xu L. L. Chan K. M. (2013). Engineered scaffold-free tendon tissue produced by tendon-derived stem cells. *Biomaterials* 34 2024–2037. 10.1016/j.biomaterials.2012.11.046 23246065

[B29] Peffers M. J. Fang Y. Cheung K. Wei T. K. Clegg P. D. Birch H. L. (2015). Transcriptome analysis of ageing in uninjured human achilles tendon. *Arthritis Res. Ther.* 17:33. 10.1186/s13075-015-0544-2 25888722 PMC4355574

[B30] Popov C. Kohler J. Docheva D. (2015). Activation of EphA4 and ephb2 reverse signaling restores the age-associated reduction of self-renewal, migration, and actin turnover in human tendon stem/progenitor cells. *Front. Aging Neurosci.* 7:246. 10.3389/fnagi.2015.00246 26779014 PMC4701947

[B31] Pryce B. A. Watson S. S. Murchison N. D. Staverosky J. A. Dunker N. Schweitzer R. (2009). Recruitment and maintenance of tendon progenitors by TGFbeta signaling are essential for tendon formation. *Development* 136 1351–1361. 10.1242/dev.027342 19304887 PMC2687466

[B32] Rahmati M. Nalesso G. Mobasheri A. Mozafari M. (2017). Aging and osteoarthritis: central role of the extracellular matrix. *Ageing Res. Rev.* 40 20–30. 10.1016/j.arr.2017.07.004 28774716

[B33] Rees J. D. Wilson A. M. Wolman R. L. (2006). Current concepts in the management of tendon disorders. *Rheumatology* 45 508–521. 10.1093/rheumatology/kel046 16490749

[B34] Rentz T. J. Poobalarahi F. Bornstein P. Sage E. H. Bradshaw A. D. (2007). SPARC regulates processing of procollagen I and collagen fibrillogenesis in dermal fibroblasts. *J. Biol. Chem.* 282 22062–22071. 10.1074/jbc.M700167200 17522057

[B35] Schiele N. R. Marturano J. E. Kuo C. K. (2013). Mechanical factors in embryonic tendon development: potential cues for stem cell tenogenesis. *Curr. Opin. Biotechnol.* 24 834–840. 10.1016/j.copbio.2013.07.003 23916867 PMC3813901

[B36] Schneider M. Angele P. Jarvinen T. A. H. Docheva D. (2018). Rescue plan for achilles: therapeutics steering the fate and functions of stem cells in tendon wound healing. *Adv. Drug Deliv. Rev.* 129 352–375. 10.1016/j.addr.2017.12.016 29278683

[B37] Screen H. R. Berk D. E. Kadler K. E. Ramirez F. Young M. F. (2015). Tendon functional extracellular matrix. *J. Orthop. Res.* 33 793–799. 10.1002/jor.22818 25640030 PMC4507431

[B38] Shen H. Jayaram R. Yoneda S. Linderman S. W. Sakiyama-Elbert S. E. Xia Y. (2018). The effect of adipose-derived stem cell sheets and CTGF on early flexor tendon healing in a canine model. *Sci. Rep.* 8:11078. 10.1038/s41598-018-29474-8 30038250 PMC6056475

[B39] Smith R. K. Birch H. L. Goodman S. Heinegard D. Goodship A. E. (2002). The influence of ageing and exercise on tendon growth and degeneration–hypotheses for the initiation and prevention of strain-induced tendinopathies. *Comp. Biochem. Physiol. A Mol. Integr. Physiol.* 133 1039–1050. 10.1016/s1095-6433(02)00148-4 12485691

[B40] Sousa-Victor P. Gutarra S. Garcia-Prat L. Rodriguez-Ubreva J. Ortet L. Ruiz-Bonilla V. (2014). Geriatric muscle stem cells switch reversible quiescence into senescence. *Nature* 506 316–321. 10.1038/nature13013 24522534

[B41] Steinmann S. Pfeifer C. G. Brochhausen C. Docheva D. (2020). spectrum of tendon pathologies: triggers, trails and end-state. *Int. J. Mol. Sci.* 21:844. 10.3390/ijms21030844 32013018 PMC7037288

[B42] Stolzing A. Scutt A. (2006). Age-related impairment of mesenchymal progenitor cell function. *Aging Cell* 5 213–224. 10.1111/j.1474-9726.2006.00213.x 16842494

[B43] Svensson R. B. Heinemeier K. M. Couppe C. Kjaer M. Magnusson S. P. (2016). Effect of aging and exercise on the tendon. *J. Appl. Physiol.* 121 1237–1246. 10.1152/japplphysiol.00328.2016 27150831

[B44] Tan G. K. Pryce B. A. Stabio A. Brigande J. V. Wang C. Xia Z. (2020). Tgfbeta signaling is critical for maintenance of the tendon cell fate. *eLife* 9:e52695. 10.7554/eLife.52695 31961320 PMC7025861

[B45] Tarafder S. Gulko J. Kim D. Sim K. H. Gutman S. Yang J. (2019). Effect of dose and release rate of CTGF and TGFbeta3 on avascular meniscus healing. *J. Orthop. Res.* 37 1555–1562. 10.1002/jor.24287 30908692 PMC6601329

[B46] Taylor R. W. Barron M. J. Borthwick G. M. Gospel A. Chinnery P. F. Samuels D. C. (2003). Mitochondrial DNA mutations in human colonic crypt stem cells. *J. Clin. Investig.* 112 1351–1360. 10.1172/jci19435 14597761 PMC228466

[B47] Theocharis A. D. Skandalis S. S. Gialeli C. Karamanos N. K. (2016). Extracellular matrix structure. *Adv. Drug Deliv. Rev.* 97 4–27. 10.1016/j.addr.2015.11.001 26562801

[B48] Turlo A. J. Ashraf Kharaz Y. Clegg P. D. Anderson J. Peffers M. J. (2018). Donor age affects proteome composition of tenocyte-derived engineered tendon. *BMC Biotechnol.* 18:2. 10.1186/s12896-018-0414-5 29338716 PMC5771075

[B49] Yan Z. Yin H. Nerlich M. Pfeifer C. G. Docheva D. (2018). Boosting tendon repair: interplay of cells, growth factors and scaffold-free and gel-based carriers. *J. Exp. Orthop.* 5:1. 10.1186/s40634-017-0117-1 29330711 PMC5768579

[B50] Yin H. Caceres M. D. Yan Z. Schieker M. Nerlich M. Docheva D. (2019). Tenomodulin regulates matrix remodeling of mouse tendon stem/progenitor cells in an ex vivo collagen I gel model. *Biochem. Biophys. Res. Commun.* 512 691–697. 10.1016/j.bbrc.2019.03.063 30922565

[B51] Yin H. Strunz F. Yan Z. Lu J. Brochhausen C. Kiderlen S. (2020). Three-dimensional self-assembling nanofiber matrix rejuvenates aged/degenerative human tendon stem/progenitor cells. *Biomaterials* 236:119802. 10.1016/j.biomaterials.2020.119802 32014804

[B52] Yin Z. Guo J. Wu T. Y. Chen X. Xu L. L. Lin S. E. (2016). stepwise differentiation of mesenchymal stem cells augments tendon-like tissue formation and defect repair in vivo. *Stem Cells Transl. Med.* 5 1106–1116. 10.5966/sctm.2015-0215 27280798 PMC4954446

[B53] Yu J. M. Wu X. Gimble J. M. Guan X. Freitas M. A. Bunnell B. A. (2011). Age-related changes in mesenchymal stem cells derived from rhesus macaque bone marrow. *Aging Cell* 10 66–79. 10.1111/j.1474-9726.2010.00646.x 20969724 PMC4339051

[B54] Zhang H. Menzies K. J. Auwerx J. (2018). The role of mitochondria in stem cell fate and aging. *Development* 145:dev143420. 10.1242/dev.143420 29654217 PMC5964648

