# Supplementary material for: Aged Tendon Stem/Progenitor Cells Are Less Competent to Form 3D Tendon Organoids Due to Cell Autonomous and Matrix Production Deficits
Source: Front Bioeng Biotechnol. 2020 May 5;8:406. doi: 10.3389/fbioe.2020.00406 (PMC7214752; doi:10.3389/fbioe.2020.00406)
Supplement: Supplementary file 1 [file Table_1.DOCX]

**Supplement table. Summary of the donor groups.**

| **Donor groups** | **Clinical indications** | **Histological examination**** | **Inclusion criteria** | **Exclusion Criteria** |
| --- | --- | --- | --- | --- |
| young/healthy  (N = 4; Mean age = 28±5) 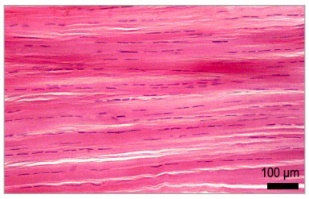 | 1. Accidents of the lower extremity with intact Achilles tendon;  2. Surgical procedure:  Amputation | 1. Degenerative lesions: Macroscopic findings (thicker, softer, yellowish, lost luster);  2. Histological findings  (1) structural anomalies: loss of normal pattern of collagen fiber alignment, areas of stromal homogenization due to hyalinisation;  (2) loss of collagen stainability characterized by pale collagen fiber staining;  (3) cellular changes:  rounded nuclei, increased cellularity, ingrowth of vessels. | 1. Donor consent;  2. Surgical indications for lower extremity operation;  3. Tissue not needed for further surgical procedures;  4. Intact Achilles tendon;  5. Males; | 1. Tendon rupture;  2. Tendon abrasion;  3. Chronic or local infections;  4. Tendonitis;  5. Tumor patients;  6. Systemic or local glucocorticosteroid treatment;  7. Autoimmune diseases (rheumatic diseases, collagenoses);  8. Females (due to unknown hormonal effects) |
| aged/degenerated  (N = 12; Mean age = 63±14*)  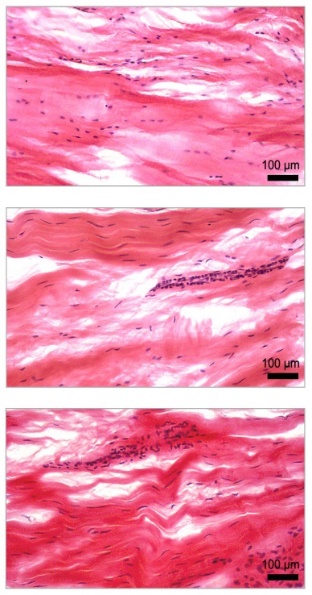 | 1. Deformities of ankle or mid-foot joints (osteoarthritis, arthropathy);  2. Surgical procedure:  Arthrodesis with tenotomy of the Achilles tendon |  |  |  |

* One 37 years old donor was included in this group because of clear macroscopic and histological signs of Achilles tendon matrix degeneration.

** According to Puddu G, Ippolito E, Postacchini F (1976). A classification of achilles tendon disease. Am J Sports Med. **4**, 145-150.
